# Indicators to measure risk of disaster associated with drought: Implications for the health sector

**DOI:** 10.1371/journal.pone.0181394

**Published:** 2017-07-25

**Authors:** Aderita Sena, Kristie L. Ebi, Carlos Freitas, Carlos Corvalan, Christovam Barcellos

**Affiliations:** 1 Institute of Scientific and Technological Communication and Information in Health (ICICT), Oswaldo Cruz Foundation (Fiocruz), Rio de Janeiro, Brazil; 2 Department of Global Health, University of Washington, Seattle, Washington, USA; 3 National School of Public Health (ENSP), Oswaldo Cruz Foundation (Fiocruz), Rio de Janeiro, Brazil; 4 Faculty of Health, University of Canberra, Canberra, Australia; University of Vermont, UNITED STATES

## Abstract

**Introduction:**

Brazil has a large semiarid region, which covers part of 9 states, over 20% of the 5565 municipalities in the country and at 22.5 million persons, 12% of the country’s population. This region experiences recurrent and extended droughts and is characterized by low economic development, scarcity of natural resources including water, and difficult agricultural and livestock production. Local governments and communities need easily obtainable tools to aid their decision making process in managing risks associated with drought.

**Methods:**

To inform decision-making at the level of municipalities, we investigated factors contributing to the health risks of drought. We used education and poverty indicators to measure vulnerability, number of drought damage evaluations and historical drought occurrences as indicators of hazard, and access to water as an indicator of exposure, to derive a drought disaster risk index.

**Results:**

Indicators such as access to piped water, illiteracy and poverty show marked differences in most states and, in nearly all states, the living conditions of communities in the semiarid region are worse than in the rest of each state. There are municipalities at high drought disaster risk in every state and there are a larger number of municipalities at higher risks from the center to the north of the semiarid region.

**Conclusions:**

Understanding local hazards, exposures and vulnerabilities provides the means to understand local communities’ risks and develop interventions to reduce them. In addition, communities in these regions need to be empowered to add their traditional knowledge to scientific tools, and to identify the actions most relevant to their needs and realities.

## Introduction

The interaction between climatic, environmental and social factors influences the conditions of vulnerability at the local level, and these in turn are modified depending on socioeconomic, cultural and political forces in place [1–6]. The magnitude of the impacts of extreme weather and climate events depend on the level of vulnerability and exposure of a community to these events. Therefore, these two elements are established as driving forces to reduce disaster risks and their impacts and increase resilience [5,7,8].

Droughts occur slowly and silently, without causing short-term impacts, making their timely identification difficult. Their multiple health impacts are often not recognized, but they include different health outcomes such as water-borne diseases, vector-borne disease, nutritional problems, mental health conditions, and respiratory diseases [9–11]. This invisibility also makes it difficult to identify when a drought starts and stops [2,12]. These characteristics classify drought into four different types, depending on the time scale and on the stakeholders’s perspective, which are defined as: meteorological, agricultural (or soil moisture drought), hydrological and socioeconomic drought. Meteorological drought is a natural event that results from a period with an abnormal precipitation deficit; agricultural drought is defined as a shortage of precipitation during the growing season that causes deficit of soil moisture, which causes several effects on agroecosystems; hydrological drought refers to negative effects of periods of precipitation shortfalls on surface or subsurface water supply (anomalies in stream flow, and in the levels of reservoir, lake, groundwater); and socioeconomic drought occurs when the demand of some economic goods (water, food grains, fish, hydroelectric power) exceeds supply due to a weather-related deficit in water supply [13]. The impacts of droughts are the result of the physical nature of the hazard and the affected communities’ ability to manage the risks [14]. In regions where there are recurrent and prolonged drought events, environmental vulnerabilities are added to existing socioeconomic vulnerabilities, and to weak infrastructure and political organization, increasing impacts on the communities [2,3,14]. A better understanding of these interactions is valuable in order to map risks and vulnerabilities, and to identify the possible impacts and needs. These, in turn, will aid in risk management and adaptation measures before and during a drought, and aid in establishing measures to prevent and/or reduce future risks [1,5,8,15–18].

Inequalities and social injustice can create or amplify the degree of vulnerability and the proportion of persons living in duress, producing an unjust cycle of perpetuation of poverty and vulnerability [2,10,19,20]. These pressures can cause temporary or permanent migration processes, generating other pressures and problems both to those who migrated and those who were left behind, limiting access to essential resources for life [2,7,11,12,18,21,22]. Sustainable development and adaptation strategies can help reduce exposure, vulnerabilities and risks, and can help build capacity and resilience of communities, government, as well as protect fragile ecosystems that support the livelihood of the affected communities [1,2,8,17,20,23,24].

**Fig 1:**
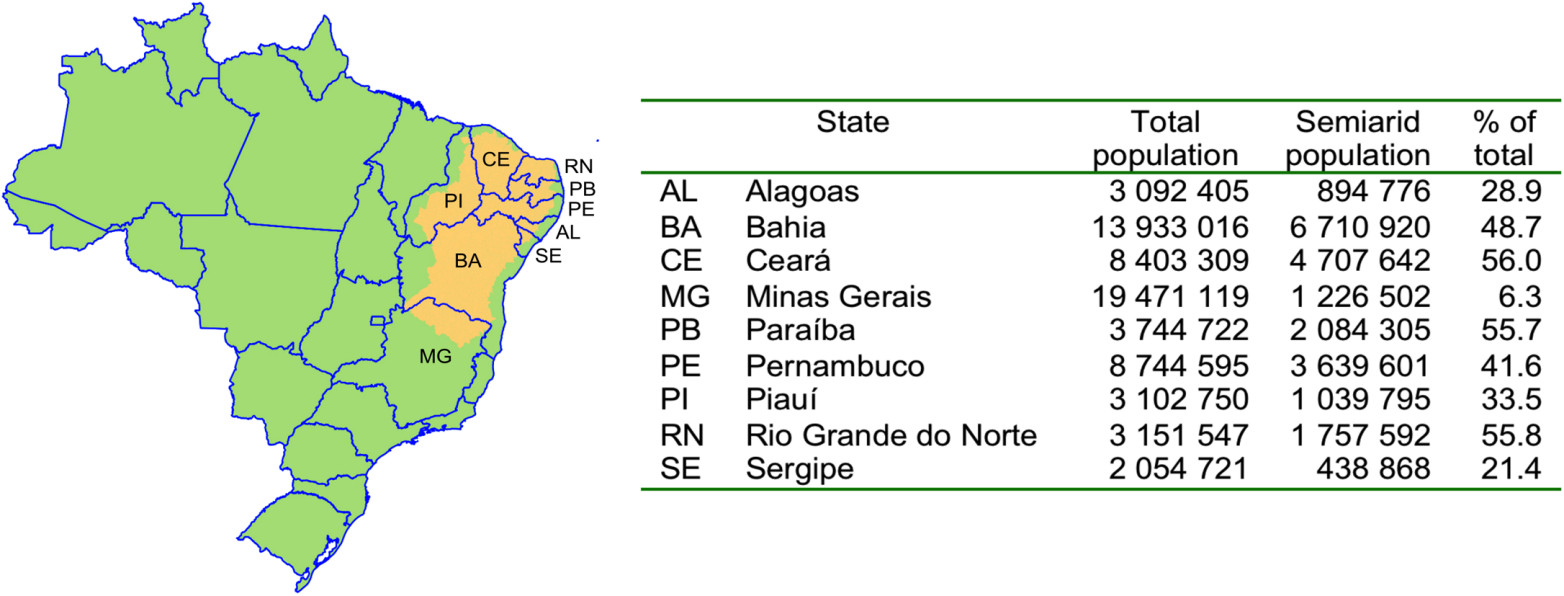
Population living in the semiarid region of 9 Brazilian states. Source: Adapted from references [26,27,28].

Brazil has a large semiarid region, which covers part of 9 states and includes 1,135 municipalities (just over 20% of the 5,565 municipalities in the country). The region is home to approximately 22.5 million persons, or about 34% of the population in the 9 states, and almost 12% of the population of Brazil [25,26], (Fig 1 [26–28]). The Brazilian semiarid region shows recurrent and extended droughts, and the area is characterized by low economic development, scarcity of natural resources (including water), and difficult agricultural and livestock production. These characteristics have negative impacts on the living conditions of the communities that live in this region [25,29].

Human activities also impact on land degradation, which contributes to the desertification process, drought severity, increasing food insecurity, poverty and inequality [29,30]. Therefore, ensuring food security, access to water, access to health care, combating desertification, land degradation and overuse of natural resources, and the reduction of risks, vulnerabilities and impacts from drought related disasters are key to achieving sustainable and equitable growth and development in communities living in drought prone areas.

### Vulnerability and resilience

Disaster is defined by the Intergovernmental Panel on Climate Change (IPCC) as alterations in the normal functioning of a community or a society due to hazardous physical events, which interact with vulnerable social conditions [2]. The disaster risk is the likelihood that a disaster could occur, which derives from a combination of physical hazards and the existing vulnerabilities [7,22]. Disaster risk management, which is of particular interest to health decision-makers, should focus on reducing exposure and vulnerability and in increasing resilience to potential adverse effects of a climate related extreme, such as drought [2].

The term “vulnerability” is used in diverse disciplines, with similar meaning, which varies according to the field in question [5,31]. Generically, vulnerability is seen as the level of probability of a system or population, to suffer damages from exposure to a threat, a change in the environment, or a social stress associated to social, economic and environmental factors [20,32–34]. Vulnerability is defined by the IPCC as the propensity or predisposition to be adversely affected [2]. Vulnerability is also determined by the lack of capacity to cope, respond and adapt to different stresses [5,15,35–38]. The World Health Organization (WHO) adopts the IPCC definition of vulnerability to climate change, as the degree to which populations, systems and places are susceptible or incapable to deal with adverse impacts, and being influenced by a variety of other factors (physical, biological, social, cultural, economic, political, institutional, and access or control over resources for subsistence) [2,38]. When health problems coexist, vulnerability to other risks increases, making the initial health conditions worse [5,39–41].

Earlier studies understand vulnerability, in the context of climate change, as a function of three related dimensions: exposure, sensitivity and adaptive capacity. Exposure involves the intensity, frequency, duration and spatial extension of threats to populations, ecosystems and infrastructures. Sensitivity can be understood as the life conditions of a population, which can modify how individuals respond to exposures (e.g. biological or socioeconomic conditions) [42]. Adaptive capacity includes social and economic determinants, together with choices and opportunities to resist to or reorganize after a stress or shock [43]. The Special Report of the IPCC on Managing the Risks of Extreme Events and Disasters to Advance Climate Change Adaptation (SREX) [2] adopts instead the concept of risk, as the result from the interaction of three dimensions: Hazard (climate and weather events); Exposure; and Vulnerability. That report shows high confidence that the severity of the impacts from extreme climate events depend on the degree to which socio-ecological systems are exposed and existing social vulnerabilities. Therefore, these elements are key to establishing actions in adaptation and disaster risk management [2,7,8].

A related term often linked to vulnerability is “resilience”. In social sciences and related fields resilience is defined in similar terms as the capacity of populations, places or systems to anticipate, absorb, accommodate, or recover from the effects of an event or adverse situation, while maintaining its basic functions and structures, its capacity to adapt, learn and transform itself [2,3,7,35,38,44–46].

**Fig 2:**
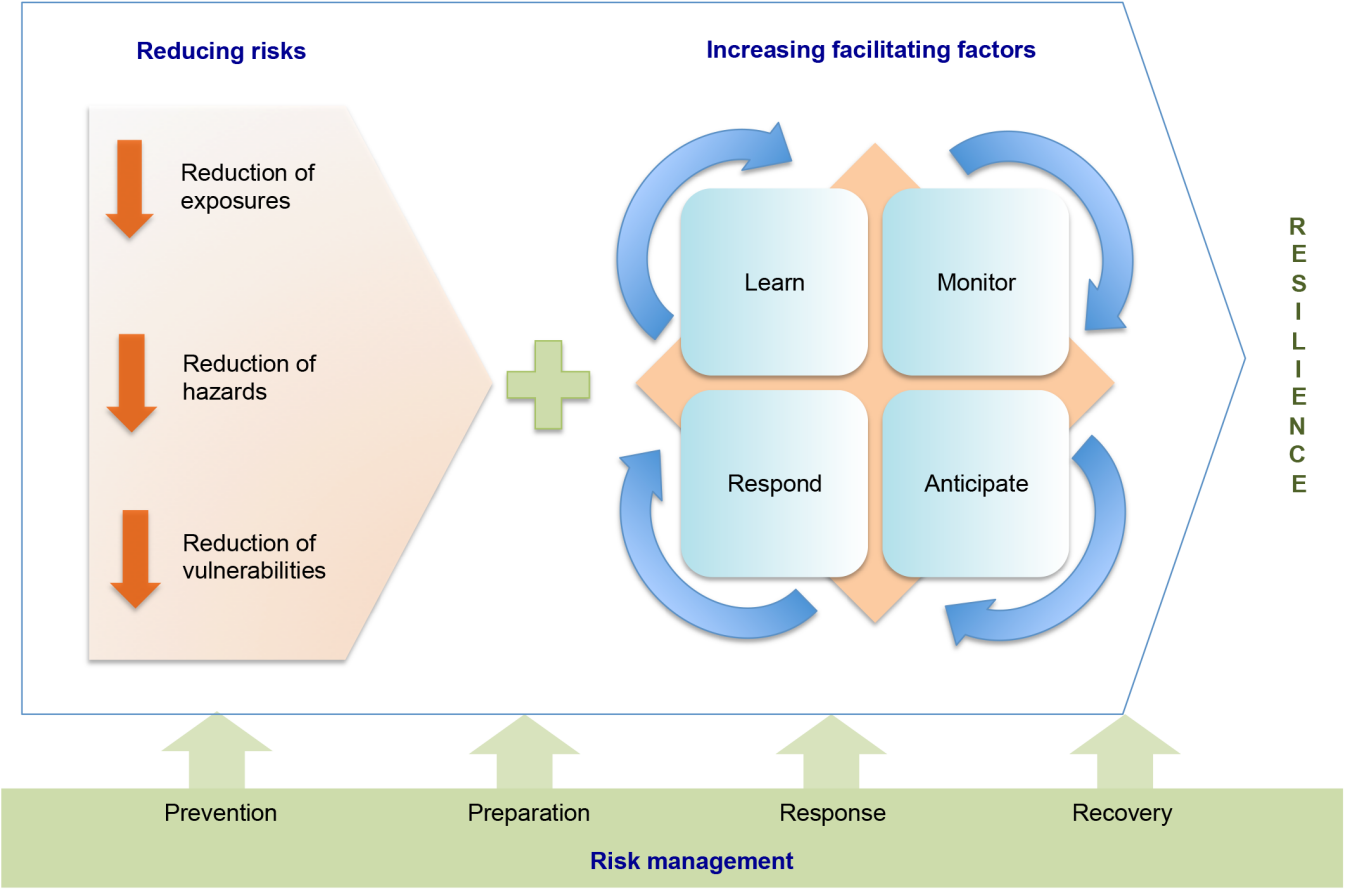
Process of building community resilience. Source: Based on the concepts of risk and risk management [2], and resilience [38,50–52].

According to Hollnagel et al. [47], resilience is supported by four pillars, or essential capacities needed for a person, community or system to be resilient: learn, monitor, anticipate and respond. This process of constructing resilience increases the capacity of adaptation and response, and can be applied to populations or systems (such as local health systems) as shown in Fig 2 [2,38,48–52].

Within the stages of facilitating factors in Fig 2, learning refers to the capacity to recognize risks and vulnerabilities with the objective of improving preparation and responses in front of an event. Monitoring is essential to the assessment of established actions. Anticipating is a proactive action, which supports prevention measures, and helps in the stages of preparation and response within the risk management framework. Responding means to attend promptly to the basic and immediate needs of exposed or affected populations. This last stage requires the three earlier stages of learning, monitoring and anticipating [17,49,50,53,54].

In the health sector, a climate resilient health system is one that is capable to anticipate, respond to, cope with, recover from, and adapt to climate-related shocks and stresses. This requires several important policies and measures of disaster risk management (prevention, preparation, response and recovery) as follows: a) prevent by recognizing, monitoring, anticipating, communicating changes needed to manage disaster risk and reduce risks to health; b) prepare, including the response, management and confronting adversities and stress in an integrated manner; c) respond, including assessing and adapting processes to change the conditions of risk and the responses required; and d) recover from the crisis with minimum negative impacts. The whole process is completed with a cycle of learning from the experience to develop further response capacity for future events [38,52], along with adaptive management, innovation, leadership, and community participation [7,10,55].

To ensure building climate resilience in the health sector and to provide a comprehensive health response, the WHO proposes actions based on the essential public health services, in six components that need to be strengthened. These are 1) leadership and governance; 2) health workforce; 3) health information systems (which includes vulnerability, capacity and adaptation assessment; integrated risk monitoring and early warning; and health and climate research); 4) essential medical products and technologies (including climate resilient and sustainable technologies and infrastructure); 5) service delivery (which includes management of environmental determinants of health; climate-informed health programmes; emergency, preparedness and management); and 6) climate and health financing [38].

Local governments and communities need easily obtainable tools to aid their decision making process in managing risks, in particular health risk, in front of recurrent droughts. The development of indicators based on existing data would be a useful tool to ensure the implementation of priority actions to prevent adverse health impacts by understanding existing hazards, reducing vulnerabilities and exposures, in order to minimize risks and their impacts.

## Methods

We applied the concept of disaster risk, as a function of vulnerability, hazard and exposures as proposed by the SREX [2] at the level of municipality. We aimed at using easily obtainable indicators from Census data (available for 1991, 2000 and 2010) [27], and chose comparable indicators to construct the components of risk using simple formulae (arithmetic means). We constructed a vulnerability index based on two variables, poverty and educational level at the municipal level. Poverty is measured as the proportion of persons living with less than BRL 140 per month (approximately USD 80 on 1 August 2010). The proportion of poor in the 1,135 municipalities ranged from 9.8% to 47.4%. Educational level is measured as the proportion of illiterate persons aged over 18 years. The proportion of illiterate persons in the region ranged from 9.8% to 67.9%. We assumed equal weight for the two variables, and the theoretical limits are 0 to 100. The index was constructed as:

V=(p+e)/2

Where V = vulnerability; p = poverty; e = educational level

We constructed a hazard index based on 2 variables: Number of drought damage evaluations and drought incidence. Damage evaluations were computed as the number of times each municipality had issued an evaluation of damages report (pre-condition to determine a state of drought). Data are available from the Integrated Information System on Disasters [56], a database that includes all types of disasters in Brazil from 1940 to December 2015. The database divides drought into two types, one of short duration called “*estiagem*” (disaster code 14110) and drought per se, with long time duration, called “*seca*” (disaster code14120). We searched for all reported damage evaluations in their initial form called “*AVADAN*”, and in their current form called “*FIDE*”. We searched for data from 1 January 1990 to 31 December 2015, and obtained the number of events for each municipality. In the 15-year period, municipalities registered between 0 and 21 events. These events were recoded to a scale of 0 to 100. The drought incidence indicator is a geographical determination of the historical level of drought, available from the National Institute of the Semiarid in Brazil [26]. It divides the municipalities of the semiarid into 5 categories of drought occurrence ranging from 0–20% to 80–100% [29]. We used the mid value to recode the 5 categories as 10, 30, 50, 70 and 90. We then computed the hazard index as:

H=(de+di)/2

Where H = hazard; de = number of drought evaluations; di = drought incidence

For exposure we used the percentage of population living in households with piped water in its inverse form (lack of access). There are many municipalities where access to piped water is very low, and even in communities with piped water access, water is not always available. The percentage of households with piped water in the region ranges from 0.2% to 100%.

Based on the above, the risk index was computed as:

R=(V+H+E)/3

Where R = Risk; V = Vulnerability; H = Hazard; E = Exposure

Data were analyzed with the software program R [57], and the mapping was performed using QGIS software [58].

## Results

**Fig 3:**
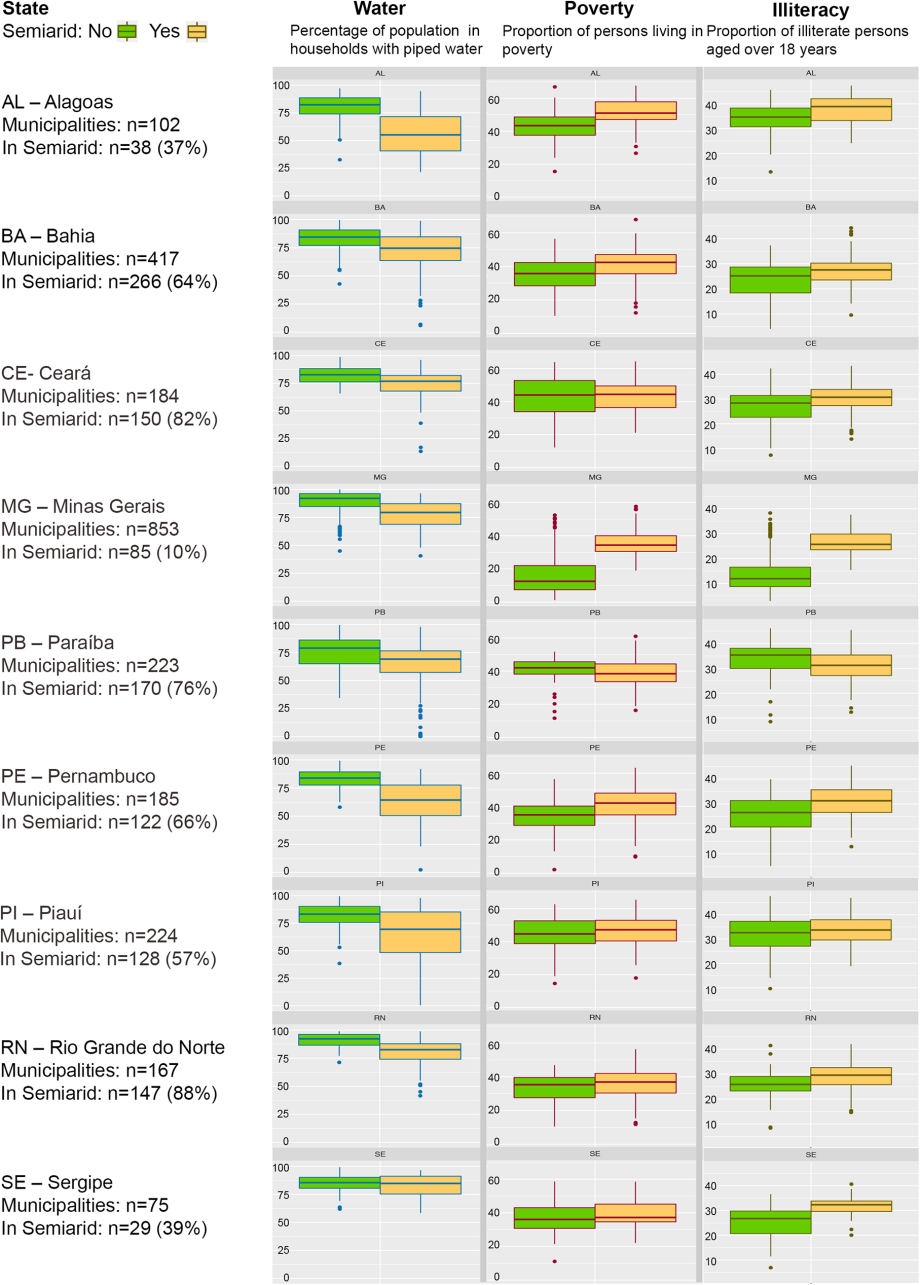
Box plots of exposure (water) and vulnerability variables (poverty and illiteracy) by State, for municipalities in and outside the semiarid region of Brazil. Source: Based on Census data from IBGE [27].

Within the 9 states, the social and environmental conditions of the population living within and outside their respective semiarid region are not equal. Indicators such as access to piped water; illiteracy and poverty show marked differences in most states. Overall, in nearly all states, the living conditions of communities in the semiarid region are worse, as shown in Fig 3 [27].

For example, access to piped water shows important differences between and within states. In all states, communities in the semiarid region have a worse situation than their counterparts outside the region. With regards to poverty and illiteracy, in all states with the exception of Paraiba, the situation is worse in communities living in the semiarid region. Minas Gerais, a state with very good indicators has very large differences between municipalities within and outside the semiarid region. With the exception of large capital cities, which are outside the semiarid region, most municipalities in the 9 states are small (median population size ranging from 5,500 to 19,300), as compared to municipalities outside the semiarid region (median population size ranging from 7,200 to 31,100).

**Fig 4:**
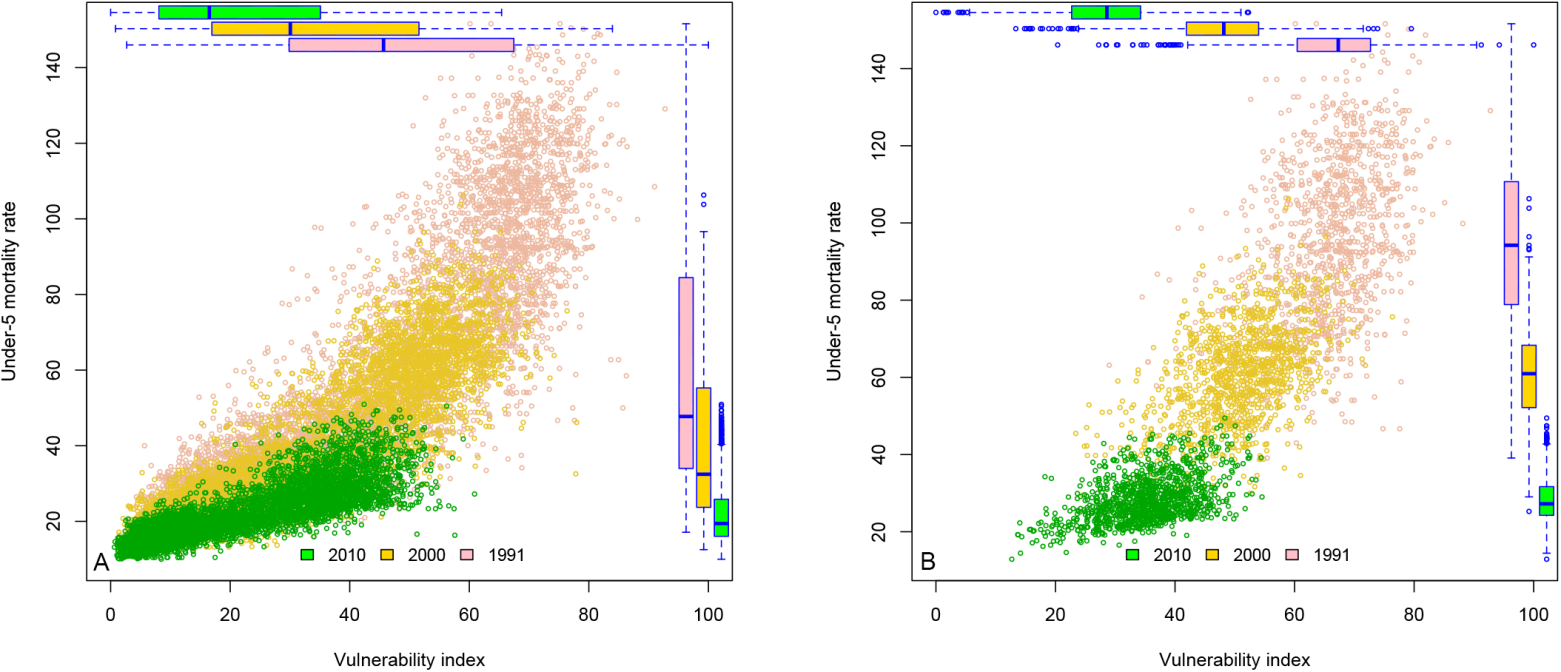
Vulnerability index by under-5 mortality rate for 1991, 2000, 2010. 4a: All 5,565 municipalities. 4b: 1,135 municipalities in the semiarid region. Source: Based on Census data from IBGE [27]

The vulnerability index based on the 2 selected indicators (poverty and illiteracy) and its association with under-5 mortality is shown in Fig 4A [27] for all 5,565 municipalities of Brazil for the years 1991, 2000 and 2010. Fig 4B [27] shows the same graph but only for the 1,135 municipalities declared as semiarid. There is a marked reduction in the vulnerability of the region between 1991 and 2010. This improvement is most prominent when applying the index to the whole country (5,565 municipalities) as shown in Fig 4A. There is also a marked improvement in the municipalities of the semiarid region, although the strong correlation between the vulnerability index and under-5 mortality for the 5,565 municipalities (r = 0.831 in 2010) is considerably reduced when analyzing only the municipalities of the semiarid (r = 0.460 in 2010). This is because the semiarid region tends to be more homogeneous in its socio-demographic characteristics, as compared to the rest of the country. However, the marked improvement in the semiarid region can be best appreciated in the box plots in Fig 4B.

**Fig 5:**
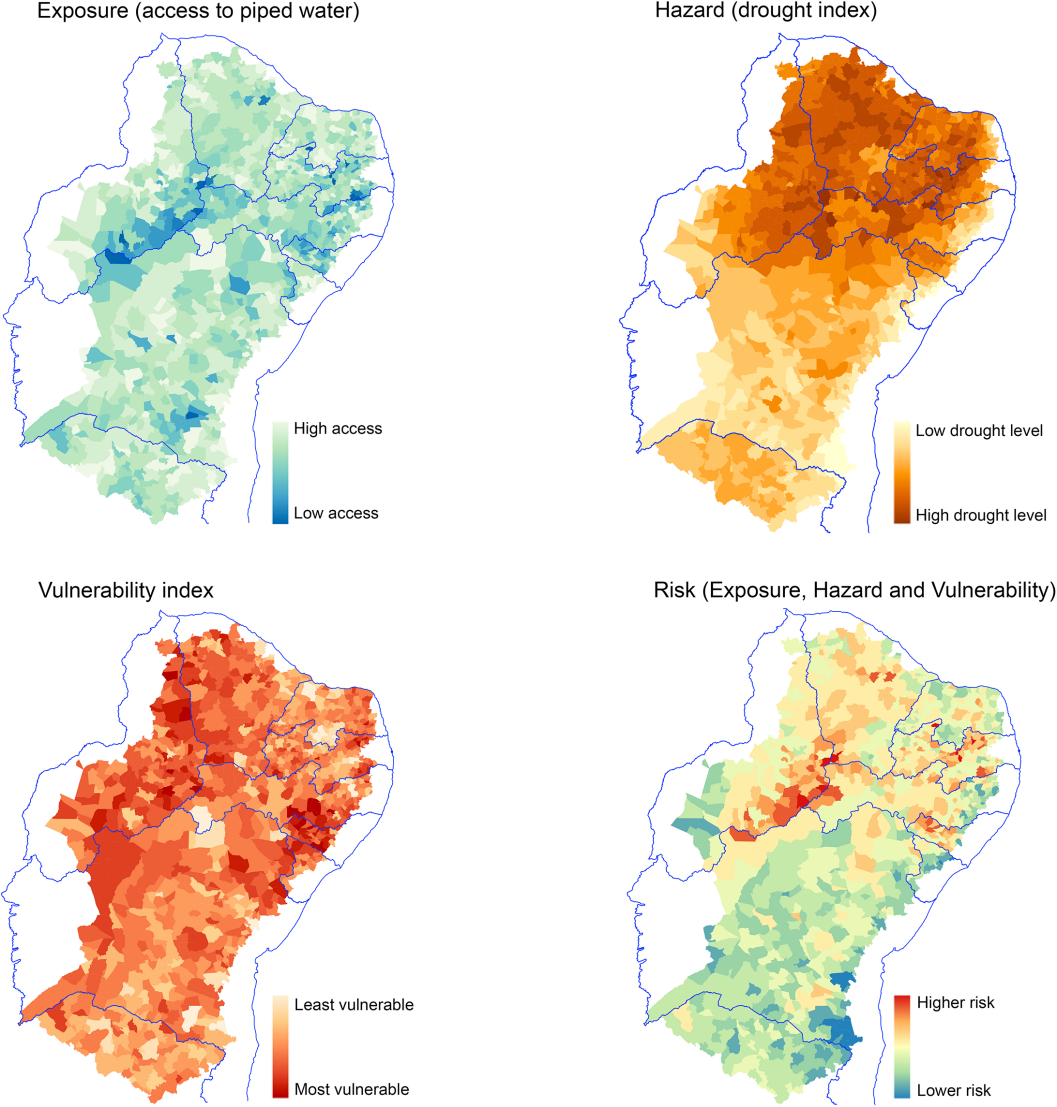
Maps of the semiarid region showing the 1,135 municipalities according to the risk index and its components. Source: based on data from [26,27,56].

The maps (Fig 5 [26,27,56]) show the risk index, and its three components (exposure, hazard and vulnerability) applied to the 1,135 municipalities of the semiarid region, for the year 2010. Highest exposures (lack of access to piped water) are more pronounced in the states of Piaui, Paraiba and Alagoas, parts of Pernambuco, and in a few municipalities in the south of Bahia. Higher drought index is observed in the municipalities of most of the 9 states, except for most of Bahia and Minas Gerais. The vulnerability index does not show clear differences among states, although parts of Bahia and Minas Gerais appear to have lower vulnerability. The combined index of risk shows a clear region of higher risk of disaster associated with drought in Piaui, and border municipalities of Pernambuco, as well as parts of Paraíba, Alagoas and Ceará. Bahia, Minas Gerais, Sergipe and Rio Grande do Norte appear to have lower overall risk of disaster associated with drought.

## Discussion

There have been a few studies that propose ways to measure vulnerability and risks, some related to climate extremes and to climate change, in Brazil [59–66]. Our study proposes a risk index of disaster associated with drought based on indicators of hazard, exposure and vulnerability, using a minimum set of highly accessible indicators, for all municipalities classified as semiarid. There are some disadvantages with this approach, as it omits details (in the form of additional indicators), which could help fine-tune the accuracy of the results. In particular, the exposure indicator (access to piped water) could be more sensitive to better measure water access, and the social costs of not having regular access. In addition, access to health care (or rather lack of) and pre-existing health conditions would be an interesting addition as modifiers of exposure (where lower access to health care or lower health status could increase the impact of exposures, such as lacking sufficient water). Although the vulnerability index (poverty and illiteracy) proposed is possibly reasonably accurate in what it intends to measure, it could be made more sensitive by adding extra variables in its construction, including on issues such as employment, and inequalities. Rapid improvement in some municipalities, as seen in Fig 4B, calls for the use of up-to-date indicators, which, although not presently available, would provide a better evolving picture of vulnerability in the lead-up to a drought. Another concern is the homogeneity of the region, which can also be observed in the vulnerability index in Fig 5. Specific to each municipality, another important dimension to add to the measurement of vulnerability is community participation in decision-making, and governance.

The hazard index is based on a well-documented geographical distribution of previous droughts and on assessments made by municipalities. The latter has the drawback that some affected municipalities may not do such assessments and lesser-affected ones would, thus biasing the index. But taking into account these caveats, the risk index and its components (vulnerability, exposure and hazard) proposed here, is a tool, which combined with other tools and strategies, could be useful in support of local decision-making and to identify actions needed at the municipal level to reduce risks and increase community resilience. This is particularly relevant in the Brazilian semiarid region, where climate change is expected to worsen the current situation (hazards) [2,67–70], and therefore reinforced action would be needed to reduce exposures and vulnerabilities.

In 2015, the Third United Nations Conference on Disaster Risk Reduction was held in Sendai, Japan. This Conference adopted the Sendai Framework for Disaster Risk Reduction 2015–2030 [55]. Table 1 [2,8,10,12,15,16,18,19,38,49,55,71–75] lists drought and health relevant actions required for reducing risks of disaster from droughts from the point of view of disaster risk management under the four priorities proposed by the Disaster Risk Reduction Framework. The risk index and its components would be a useful tool to address some of the priority areas related to health identified in this table.

**Table 1:** Examples of health sector priority measures to address disaster risk management for risks associated to drought.

Priority 1—Understanding of disaster risk
a) To map and assess the exposures and vulnerabilities of social, ecological, geographical, economic, cultural and political systems, including health services at the local level to recognize the existing risks; b) To know and integrate information about the process of climate change and climate variability and its local impacts in the context of event-specific hazard-exposure and vulnerability information to assess risks to health; c) To develop and implement local strategies to strengthen public education and awareness in health risk reduction, including drought disaster risk information and communication, through campaigns, social media and community mobilization; d) To know the health situation of the communities in particular health determinants related to drought, in order to identify indicators to facilitate relevant and timely action.
Priority 2—Strengthening disaster risk governance to manage disaster risk
a) To develop and promote the incorporation of disaster risk management plans in actions planned jointly by communities and health sector professionals. b) To implement tools to assess the degree of vulnerability, risks and threats that influence human health, from the point of view of increasing capacity of the social, political, environmental and economic systems and health services; c) To facilitate and support local multisectoral cooperation among health professionals in local governments that have a very similar situation as identified by indicators of exposure, hazard, vulnerability and risk.
Priority 3—Investing in disaster risk reduction for resilience
a) To promote and implement measures for disaster risk resilience of communities including on health information, awareness and education; b) To strengthen the implementation of policies and plans, to manage risks before, during and after drought disasters including through community involvement, and access to basic health-care services; c) To enhance the resilience of national health systems, including by integrating disaster risk management into primary, secondary and tertiary health care; developing the capacity of health workers in understanding disaster risk and applying and implementing disaster risk reduction approaches in health work.
Priority 4—Enhancing disaster preparedness for effective response and to “Build Back Better” in recovery, rehabilitation and reconstruction
a) To establish a mechanism of case registry and a database of mortality and morbidity caused by drought in order to improve the prevention of adverse health impacts; b) To promote the incorporation of disaster risk management into the health sector to develop capacities that reduce disaster risk in the short, medium and long term and to ensure effective and operational response during and after disasters; c) To develop community based multi-hazard forecasting and early warning systems and disaster risk and emergency communications mechanisms, social technologies and hazard-monitoring telecommunications systems.

Adapted from references [2,8,10,12,15,16,18,19,38,49,55,71–75]

Another useful implementation of this approach would be to help improve confidence and participation of communities in health risk management, and in response to situations and impacts associated with drought. Municipalities and communities could develop their own risk index, based on their knowledge and needs, in order to identify actions to reduce risks. The participation and social mobilization in mitigation and adaptation measures by local communities would allow the exchange of knowledge, and give value to the contributions of local communities, thus facilitating the process of action [8,10,16,76,77]. This process also stresses the importance of building future scenarios regarding the local environment and the potential risks and health impacts to further inform the process of decision-making. Some of these questions could be further studied through qualitative and quantitative research.

### Conclusions

Slow onset environmental changes, such as drought, have multiple health impacts, which are made worse by the lack of recognition of the problem and the prompt response required [9]. This invisibility challenges public policies and management in respect to drought and its health impacts. Understanding local communities’ hazards, exposures and vulnerabilities provides the means to understand their risks and develop interventions to reduce them. In addition to current, unresolved challenges, communities in the semiarid region will be affected by increased hazards as a result of climate change. This makes actions to reduce exposures and vulnerabilities even more urgent.

There is a need for greater efforts to ensure the respect to basic human rights for present and future generations in regions where communities live with an arid or semiarid climate. These rights can be guaranteed by means of protection of the environment, adaptation to on-going changes, helping communities to build resilience, and in general terms, ensuring human development in the context of the sustainable development goals [78]. In this context, health should be seen as central to achieving sustainable development in arid and semiarid regions. Communities in these regions need to be further empowered to add their traditional knowledge to scientific tools, and to identify the actions most relevant to their needs and realities.

## Supporting information

S1 File Drought risk data. S1_File.csv.
(CSV)
